# LCFF-Net: A lightweight cross-scale feature fusion network for tiny target detection in UAV aerial imagery

**DOI:** 10.1371/journal.pone.0315267

**Published:** 2024-12-19

**Authors:** Daoze Tang, Shuyun Tang, Zhipeng Fan

**Affiliations:** 1 Harbin University of Commerce, Harbin, China; 2 Heilongjiang Provincial Key Laboratory of Electronic Commerce and Information Processing, Harbin, China; Macau University of Science and Technology, MACAO

## Abstract

In the field of UAV aerial image processing, ensuring accurate detection of tiny targets is essential. Current UAV aerial image target detection algorithms face challenges such as low computational demands, high accuracy, and fast detection speeds. To address these issues, we propose an improved, lightweight algorithm: LCFF-Net. First, we propose the LFERELAN module, designed to enhance the extraction of tiny target features and optimize the use of computational resources. Second, a lightweight cross-scale feature pyramid network (LC-FPN) is employed to further enrich feature information, integrate multi-level feature maps, and provide more comprehensive semantic information. Finally, to increase model training speed and achieve greater efficiency, we propose a lightweight, detail-enhanced, shared convolution detection head (LDSCD-Head) to optimize the original detection head. Moreover, we present different scale versions of the LCFF-Net algorithm to suit various deployment environments. Empirical assessments conducted on the VisDrone dataset validate the efficacy of the algorithm proposed. Compared to the baseline-s model, the LCFF-Net-n model outperforms baseline-s by achieving a 2.8% increase in the mAP_50_ metric and a 3.9% improvement in the mAP_50–95_ metric, while reducing parameters by 89.7%, FLOPs by 50.5%, and computation delay by 24.7%. Thus, LCFF-Net offers high accuracy and fast detection speeds for tiny target detection in UAV aerial images, providing an effective lightweight solution.

## Introduction

Recent advancements in control systems and target detection technologies for unmanned vehicles have had a substantial impact on various sectors, most notably transportation [1]. Among unmanned vehicle types, Unmanned Aerial Vehicles (UAVs) have become essential assets across diverse domains, including agriculture, surveillance, disaster response, and infrastructure inspection. The unique aerial perspectives provided by UAVs, combined with their efficiency and flexibility in data collection, underscore their value in these applications. Nevertheless, the majority of state-of-the-art deep neural network architectures, including SSD [2], R-CNNs [3–6], DETRs [7–9], and YOLOs [10–19], are predominantly designed and benchmarked on manually collected image datasets, such as MS-COCO [20].

These image datasets were primarily captured manually by photographers, resulting in most images being taken from a human perspective, with a limited number featuring overhead aerial views. The targets within the images generally occupy a significant portion of the frame, and the photographs were typically selected under optimal lighting conditions to minimize issues such as glare, overexposure, and underexposure. Additionally, each image tends to include a limited number of targets, with photographers often striving to exclude extraneous objects or backgrounds unrelated to the primary subject.

In contrast, images obtained from UAVs exhibit several distinct characteristics compared to those captured manually. Firstly, the target object generally constitutes only a minor fraction of the overall image area, which can complicate target detection and analysis. Additionally, lighting conditions in UAV-captured images often vary widely; some images suffer from glare or overexposure, while others may have insufficient lighting. These images are predominantly taken from high-altitude, overhead perspectives, with few captured from ground level or elevated angles. Moreover, the backgrounds in these images are often complex, featuring numerous targets that are densely arranged and overlap significantly, which can lead to challenges in distinguishing between similar-looking objects and may induce perturbations in image processing and analytic tasks.

Beyond the unique characteristics of image data, UAV aerial target detection methods are applied in two distinct scenarios. The first scenario involves using a high-performance desktop computer or server to process image data captured by UAVs in order to ensure a high level of detection accuracy. The second scenario requires real-time processing of aerial image data using the UAV’s onboard embedded microcomputer, which is typically employed for obstacle avoidance and automated mission planning. Neural network-based target detection methods must be tailored to the specific requirements of each scenario. For desktop environments, maximizing detection accuracy is crucial, whereas in embedded environments, models must balance accuracy with limited computational and memory resources. Consequently, the neural network methods for target detection should be specifically optimized to tackle the distinct complexities inherent in UAV-captured aerial imagery, thereby fulfilling the diverse requirements of various application scenarios.

Considering the unique challenges posed by UAV aerial images and UAV target detection for target detection algorithms, this paper proposes a lightweight, efficient, and detail-enhanced LCFF-Net detection algorithm. The proposed method further explores and exploits granular detail and intrinsic features within the channel domain through techniques such as reparameterisation, shared convolution, and multi-scale feature fusion. These strategies significantly improve detection accuracy and optimize model efficiency.

The primary contributions of this paper can be succinctly categorized into three fundamental areas.

1. A novel LFECB (Lightweight Feature Extraction Convolution Block) is introduced, which serves as the foundation for the proposed LFERELAN (Lightweight Feature Extraction Reparameterised Efficient Layer Aggregation Network). This network incorporates concepts from CSPNet, GELAN, and the reparameterised convolution technique to extract features at a lower cost.
2. LR-NET and LC-FPN, both grounded in the LFERELAN architecture, were engineered to optimize the backbone and neck networks of the model. These enhancements are designed to bolster the model’s capacity for detecting minute targets in UAV aerial imagery, all while maintaining an efficient architectural structure. In addition, a LDSCD-Head (Lightweight Detail-Enhanced Shared Convolution Detection Head), is proposed to further streamline the model’s complexity.
3. A series of LCFF-Net models of varying scales to accommodate different application scenarios. These range from ultra-small models optimized for deployment on embedded devices under extreme conditions to high-precision, large-parameter models intended for desktop platforms.

The structure of the paper is as follows: Section Related work offers an extensive review of related literature. Section Methods presents a detailed explanation of the enhanced LCFF-Net detection methodology. Section Results delineates the experimental setup and parameter configurations, followed by a comprehensive series of comparative evaluations, ablation analyses, and visual comparisons to substantiate the merits of the proposed model. Section Discussion and conclusion offers a synthesis of the experimental results and explores potential avenues for future research.

## Related work

Amid ongoing advancements in deep learning-driven target detection technologies, numerous real-time detection algorithms have been introduced. Among these, the YOLO (You Only Look Once) [10] algorithm, introduced in 2015, has garnered significant attention due to its accuracy and detection speed. The algorithm achieves high efficiency, flexibility, and good generalization performance through multi-scale feature fusion and multi-level prediction, ensuring robust detection accuracy.

Jocher et al. [14] proposed YOLOv5, which introduced the CSP (Cross Stage Partial) structure and the focus module in the backbone network. Moreover, this study extended the CSP architecture of CSPNet to the neck and integrated the PAFPN (Path Aggregation Network) module to bolster the network’s feature fusion capabilities, while the original SPP (Spatial Pyramid Pooling) was substituted with the more efficient SPPF (Fast SPP) structure. Li et al. [15] developed YOLOv6, employing the Rep-PAN structure in the neck network to maintain robust multi-scale feature fusion. Furthermore, an Efficient Decoupled Head was engineered in this work to accelerate model convergence. Wang et al. [16] further advanced the series with YOLOv7 by introducing the E-ELAN (Extended Efficient Aggregation Network). This network optimally leverages model parameters by controlling the shortest and longest gradient pathways, thus augmenting the model’s ability to learn multi-scale features. Building upon YOLOv5, Jocher et al. [17] introduced YOLOv8, which features the C2f (CSP Bottleneck with 2 convolutions) fusion module in the Backbone structure. This module integrates the advantages of the ELAN and C3 fusion mechanisms. Furthermore, an anchor-free approach and Decoupled-Head architecture were employed to decouple the classification and detection heads, achieving an optimal trade-off between accuracy and model efficiency. Wang et al. [18] proposed YOLOv9, integrating PGI (Programmable Gradient Information) and a GELAN (General Efficient Layer Aggregation Network) to address the issue of information loss during the feedforward process. This approach facilitates model updates and enhances detection accuracy. In the latest iteration, YOLOv10, Wang et al. [19] introduced NMS-free training and a dual allocation strategy, which reduces inference delay and computational redundancy.

Target detection in UAV imagery poses distinct challenges, including object occlusion, background noise, and fluctuating lighting conditions [21]. Unlike conventional images, UAV aerial imagery is often captured from top-down perspectives, complicating object detection [22].

Zhang et al. [23] proposed Drone-YOLO, based on YOLOv8-l, incorporating the RepVGG heavy-parameterized convolution module as the downsampling layer. In the neck, a small-sized object detection head was added by expanding the PAFPN structure to three layers. This improvement enhances the model’s capacity to spatially localize and classify target objects, thus boosting detection accuracy. Yue et al. [24] designed the LHGNet backbone based on the HGNetv2 concept, integrating the lightweight LHG block to enhance spatial feature fusion and expand the receptive field. This study further incorporated the GSConv, LGS bottleneck, LGSCSP fusion module, and LGSneck, which synergistically enhanced detection accuracy and reinforced the model’s lightweight design, particularly for small to medium-sized targets in UAV imagery. Huang et al. [25] developed EDGS-YOLO, an improvement on YOLOv8, incorporating the DDetect detection head with deformable convolution (DCNv2) to minimize feature information loss and enhance local detail collection. The use of EMA in the neck network further increases accuracy by focusing on critical regions in the image. Additionally, the C3Ghost module, which combines GhostConv and C3 modules, effectively reduced model size without sacrificing performance. Zhao et al. [26] advanced the ITD-YOLOv8 by replacing conventional convolution in the neck structure with the lightweight AKConv, reducing model complexity. And this work introduced VoVGSCSP and CoordAtt attention mechanisms, which enhance global contextual information and multi-scale features, thus improving accuracy and robustness in detecting infrared-occluded targets in complex environments.

Contemporary UAV-based detection algorithms aim to maximize the accuracy of tiny object detection; however, they often result in increased model parameters and higher computational resource demands. In light of these challenges, this paper introduced a series of efficiently lightweight structures (including LFERELAN, LR-NET, LC-FPN, and LDSCD-Head) designed to reduce model parameters and computational resource demands while enhancing the accuracy of tiny object detection.

## Methods

**Fig 1:**
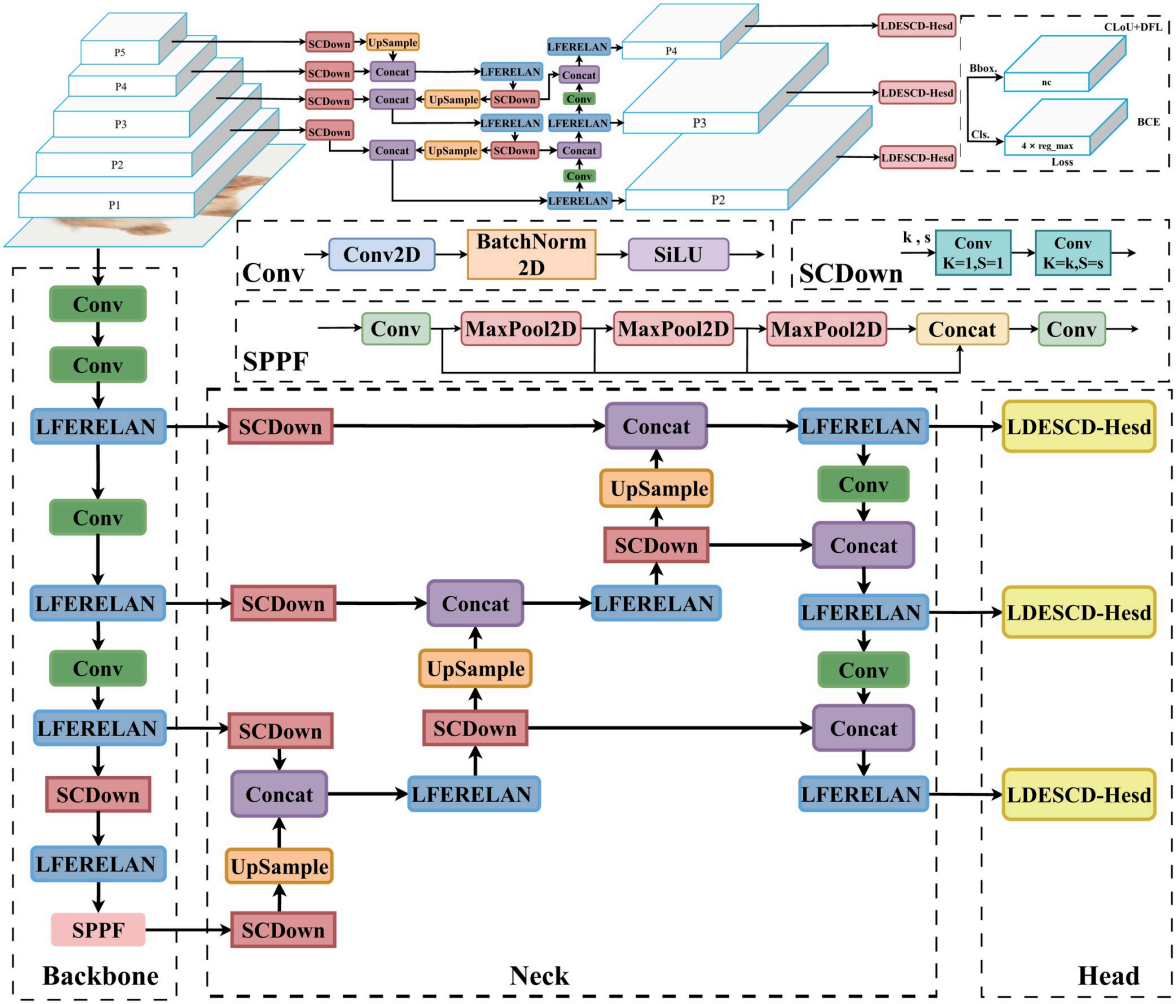
LCFF-Net network structure.

The architecture of the LCFF-Net model introduced in this paper constitutes a significant enhancement over the YOLO framework. This model employs the LR-NET, LC-FPN, and LDSCD-Head as its backbone network, neck network, and detection head, respectively; these components are introduced for the first time in this study. A detailed illustration of the LCFF-Net model’s architecture is presented in Fig 1.

Furthermore, this study refined the model’s scale configurations to develop a series of LCFF-Net models of varying scales. Significantly, the LCFF-Net-a model, distinguished by its remarkably low parameter count and computational overhead, is highly optimized for embedded deployment in resource-limited environments. This series of models demonstrated strong detection performance on the VisDrone dataset, even without employing the widely adopted attention mechanism.

### Lightweight feature extraction reparameterised efficient layer aggregation network

In the YOLO network architecture, the C2f (CSP Bottleneck with 2 Convolutions) module plays a crucial role in implementing cross-stage partial fusion. However, the bottleneck design in the C2F module incurs significant resource overhead. To mitigate this challenge, this study proposed a LFECB (Lightweight Feature Extraction Convolutional Block) that leveraged PConv (Partial Convolution) [27] and CGLU (Convolutional Gated Linear Unit) [28] to more effectively aggregated multi-scale information while minimizing resource consumption.

PConv is an advanced convolutional technique tailored to minimize computational redundancy and memory consumption by selectively applying standard convolution operations to a subset of input channels dedicated to spatial feature extraction, while preserving the remaining channels unchanged. The CGLU functions as a channel mixer that incorporates a 3 × 3 depth-wise separable convolution prior to the activation function of the GLU (Gated Linear Unit) within the gated branch. This architecture allows each token to receive a unique gating signal derived from its closest fine-grained features, thereby promoting more efficient and precise information processing. In LFECB, the output of PConv undergoes further processing through the CGLU, which optimally utilizes all channel information while minimizing computational redundancy. Additionally, DropPath is implemented following CGLU to randomly discard certain branch information, thereby enhancing the robustness of the model. Shortcut connections are also employed to improve gradient propagation and facilitate information flow.

The LFERELAN (Lightweight Feature Extraction Reparameterised Efficient Layer Aggregation Network) proposed in this paper integrates the core concepts of CSPNet [29] and GELAN [18] architectures, while also introducing the LFECB and RepConv (reparameterized convolution) [30].

LFERELAN utilizes 1×1 convolutional layers at both the input and output stages to modulate the channel dimensions. The processing results at each step occur in a branched manner: one branch directs the output immediately, while the other branch continues sequentially. Initially, the input with the adjusted number of channels undergoes a RepConv operation, followed by several cascaded LFECBs. After passing through a final 1×1 convolution, the output merges with the other branch from the preceding layers before reaching the final output.

**Fig 2:**
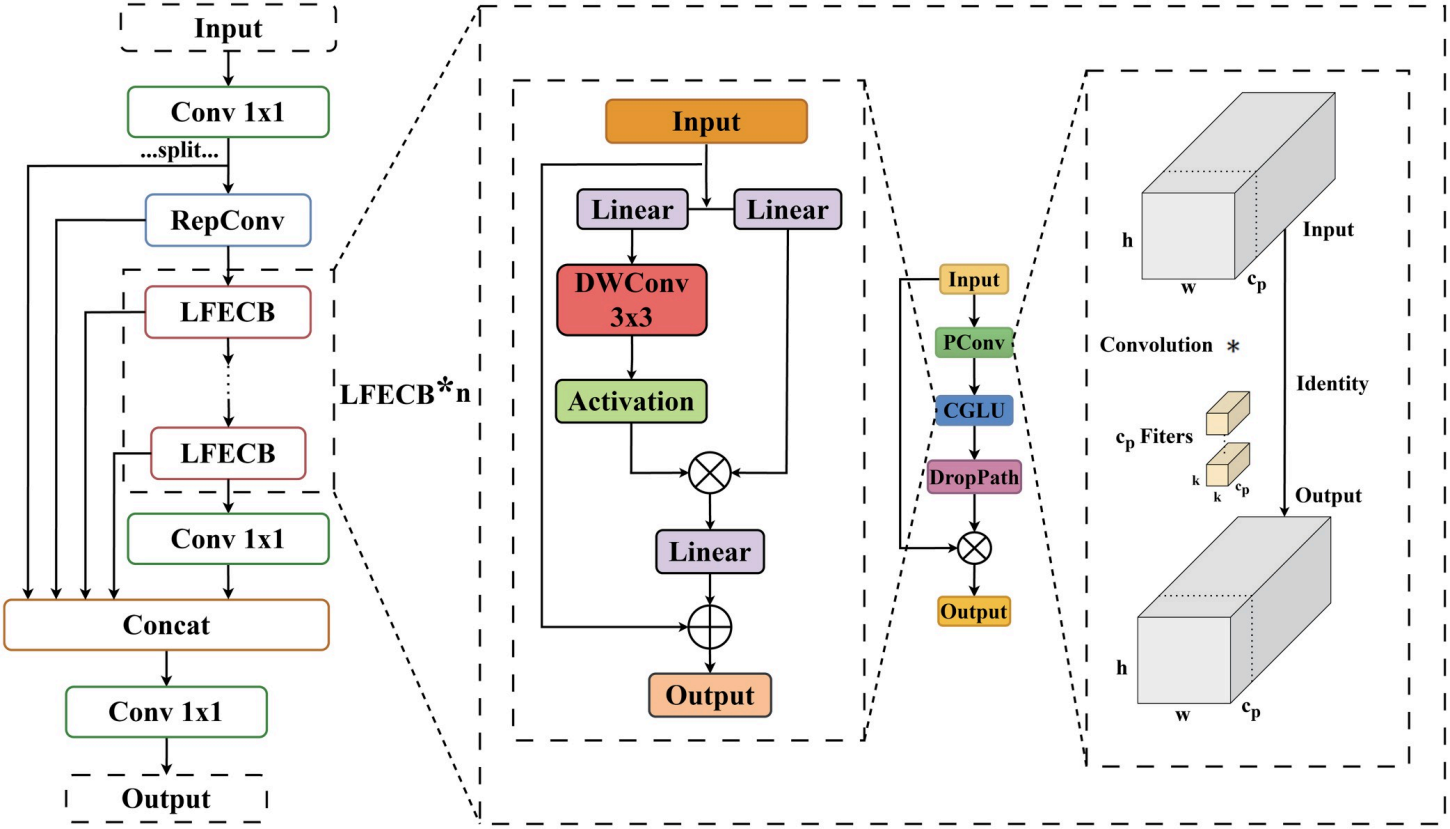
LFERELAN structure.

The core idea of the LFERELAN structural design is to reduce computational and memory overhead during inference by using RepConv to reparameterize multi-branch convolutional layers into single-branch layers, while also employing LFECB in an optimized network structure for efficient feature fusion and extraction. A comprehensive illustration of the LFECB and LFERELAN architectures is presented in Fig 2.

### Lightweight reparameterised net

LR-NET (Lightweight Reparameterised Net), designed by replacing the C2f structure with the LFERELAN structure and introducing the Spatial-channel decoupled downsampling (SCDown) [19], which consists of only two convolutional operations, building upon the original backbone architecture of YOLO, serving as the backbone network for LCFF-Net.

The following equations detail the parameter calculations for each module within LR-NET. Specifically, Eq (1) defines the parameters for the Conv module, Eq (2) for the RepConv module in the LFERELAN module, Eq (3) for the LFECB module, Eq (4) for the LFERELAN module, Eq (5) for the SCDown module, and Eq (6) for the SPPF module. In these equations, *C* denotes the number of input and output feature channels, *K* indicates the convolution kernel size used within the modules, and *n*, *E*_*x*_, and *M*_*s*_ represent the proportional parameters relevant to the LFERELAN module.

$$\begin{array}{c} Y1\left(Params_{Conv}\right)=C_{in1}C_{out1}K_{h1}K_{w1}+2C_{out1} \end{array}$$ (1)

$$\begin{array}{c} Y2\left(Params_{RepConv}\right)=10C_{in2}C_{out2}+4C_{out2} \end{array}$$ (2)

$$\begin{array}{c} Y3\left(Params_{LFECB}\right)=\frac{9C_{in3}^{2}}{16}+3C_{in3}\lfloor \frac{2}{3}C_{in3}\rfloor +12\lfloor \frac{2}{3}C_{in3}\rfloor +C_{in3} \end{array}$$ (3)

$$\begin{array}{c} Y4\left(Params_{LFERELAN}\right)=\left(4+2C_{in}+C_{out}\right)\lfloor E_{x}C_{out}\rfloor +C_{out}\left(n+1\right)\lfloor M_{s}\lfloor E_{x}C_{out}\rfloor \rfloor \\ +\lfloor M_{s}\lfloor E_{x}C_{out}\rfloor \rfloor \left(6+10\lfloor E_{x}C_{out}\rfloor \right)+2C_{out}+{\left(\lfloor M_{s}\lfloor E_{x}C_{out}\rfloor \rfloor \right)}^{2}+\left(n-1\right)\\ \left(\frac{9{\left(\lfloor M_{s}\lfloor E_{x}C_{out}\rfloor \rfloor \right)}^{2}}{16}+\lfloor \frac{2}{3}\lfloor M_{s}\lfloor E_{x}C_{out}\rfloor \rfloor \rfloor \left(12+3\lfloor M_{s}\lfloor E_{x}C_{out}\rfloor \rfloor \right)+\lfloor M_{s}\lfloor E_{x}C_{out}\rfloor \rfloor \right) \end{array}$$ (4)

$$\begin{array}{c} Y5\left(Params_{SCDown}\right)=C_{in5}C_{out5}+C_{out5}K_{h5}K_{w5}+4C_{out5} \end{array}$$ (5)

$$\begin{array}{c} Y6\left(Params_{SPPF}\right)=\frac{1}{2}C_{in6}^{2}+2C_{in6}C_{out6}+C_{in6}+2C_{out6} \end{array}$$ (6)

The core objective of LR-NET is to replace the bulkier C2f with the lighter and more efficient LFERELAN module, which significantly decreases both model parameters and computational overhead while improving performance. Additionally, the introduction of SCDown further reduces parameters and resource consumption. Consequently, the lightweight LR-NET enhances feature extraction and fusion capabilities, minimizing overall parameters and computational demands, thereby increasing its applicability for deployment in embedded systems.

**Fig 3:**
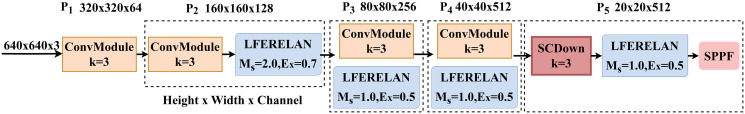
LR-NET structure.

The efficacy of LR-NET was demonstrated through the ablation study discussed in Section Ablation experiment. A detailed depiction of the LFECB and LFERELAN architectures is provided in Fig 3.

### Lightweight cross-scale feature pyramid network

The original neck structure of the YOLO model does not fully exploit the image features extracted at each layer of the backbone network. This limitation leads to suboptimal performance in certain specialised application scenarios, such as object detection in UAV imagery. To mitigate this challenge, this paper proposed a LC-FPN (Lightweight Cross-scale Feature Pyramid Network).

The LC-FPN is inspired by the CCFF (CNN-based Cross-scale Feature Fusion) [8] structure. It further adjusts and optimizes the feature fusion method and architecture. Additionally, it incorporates the LFERELAN proposed in this paper, along with SCDown. The LC-FPN is designed to function as the neck network of LCFF-Net. It first standardizes the dimensionality of image features extracted from each layer of the backbone network, followed by a hierarchical extraction and fusion of these features. Finally, downsampling is applied to output the image features at each layer for the detection head. Through this process, the information extracted by the backbone network is comprehensively utilized, particularly enhancing the detection of tiny objects by focusing on their specific feature representations.

**Fig 4:**
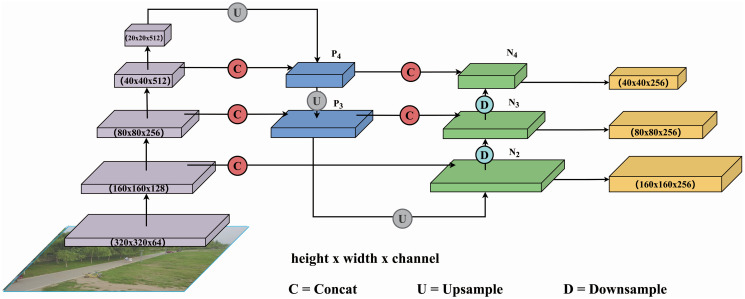
LC-FPN structure.

The effectiveness of LC-FPN has also been verified through the ablation experiment detailed in Section Ablation experiment. In comparison to the original neck architecture of the YOLOv8 model, LC-FPN notably reduces the parameter count while enhancing accuracy, particularly in detecting tiny objects within UAV aerial imagery. The LC-FPN architecture is depicted in Fig 4.

### Lightweight detail-enhanced shared convolution detection head

In the original YOLO model, three detection heads independently extract image features through two branches with two consecutive 3×3 convolutions followed by a 1×1 convolution. However, this architecture substantially inflates the model’s parameter count, with the detection head alone accounting for one-fifth of the total parameters in the entire YOLO algorithm. Furthermore, the conventional single-scale prediction structure employed by the original YOLO detection head proves insufficient for multi-scale target detection. This shortcoming arises from the model’s dependence on predictions from a single feature map scale, thus overlooking the potential contributions of multi-scale features. To mitigate this challenge, this paper proposed a novel detection head structure termed LDSCD-Head (Lightweight Detail-Enhanced Shared Convolution Detection Head).

**Fig 5:**
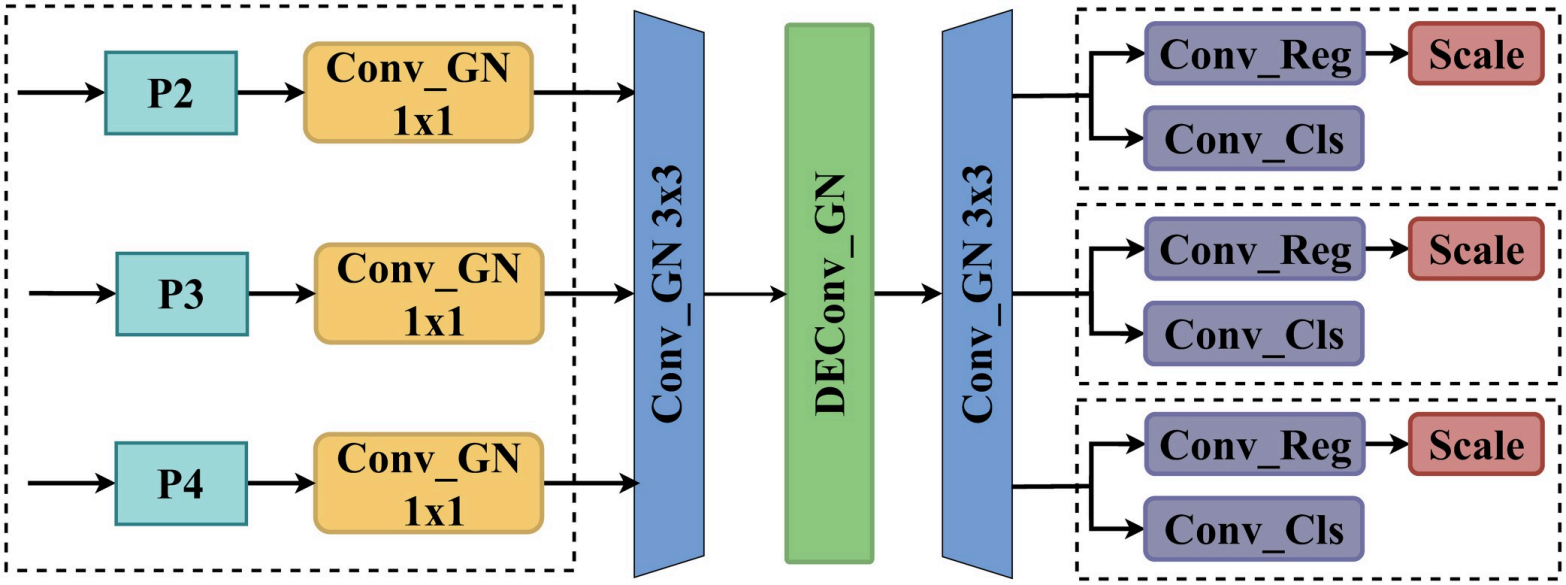
LDSCD-Head structure.

The LDSCD-Head introduces a modification to the original YOLO architecture by replacing the multiple independent convolutions of the three detection heads with a shared group-normalized DEConv (Detail-Enhanced convolution) [31] and two additional shared group-normalized convolutions, as depicted in Fig 5 (blue and green sections). To address the issue of inconsistent target scales detected by each head, a low-computation-cost scaling layer is incorporated at the input of each detection head, aligning it to a unified dimension (illustrated in the yellow section of Fig 5). This architectural adjustment significantly reduces the number of parameters while leveraging richer feature scales for target detection, thereby enhancing the multi-scale sensing capabilities of the detection heads and improving overall detection performance.

### Proposed models

To adapt the LCFF-Net algorithm to various task environments, this paper proposes several scales of the model, ranging from the smallest to the largest, denoted by configurations a, t, n, s, m, and l. These configurations are distinguished through adjustments in key architectural parameters: depth, width, and the maximum number of channels.

The depth parameter determines the number of repetitions for each feature extraction stage in the model. Increasing the depth enhances the model’s complexity and its capacity for representation; however, it also results in greater computational demand and extended inference times. The width parameter controls the number of convolutional channels within each layer, effectively setting the width of each layer’s feature space. While a larger width enables more comprehensive feature extraction at each layer, it also increases computational requirements and the total number of parameters. Finally, the maximum number of channels parameter sets an upper limit on the number of channels within each convolutional layer, ensuring that the number of channels does not exceed this specified threshold. By constraining the channel count, the model avoids excessive depth-induced channels, reducing both computational load and memory usage.

Table 1 summarizes the main differences among these configurations, including standard parameter counts and computation volumes when the model is configured for 80 input detection categories.

**Table 1:** Differences between models of different scales.

Method	Depth	Width	Max Channels	Params (M)	FLOPs (G)
LCFF-Net-a	0.33	0.12	1024	0.33	3.3
LCFF-Net-t	0.33	0.16	1024	0.64	7.1
LCFF-Net-n	0.33	0.25	1024	1.28	12.4
LCFF-Net-s	0.33	0.50	1024	5.08	47.8
LCFF-Net-m	0.40	0.75	768	9.28	104.6
LCFF-Net-l	0.45	1.00	512	14.10	184.2

Among the different scales of the LCFF-Net model, LCFF-Net-a stands out for its lightweight design and low computational requirements, while preserving a commendable level of accuracy, rendering it ideal for deployment in resource-constrained or extreme environments. Both LCFF-Net-n and LCFF-Net-t exhibit reduced parameter counts, offer satisfactory performance, and deliver faster processing speeds, making them applicable to a broader range of scenarios. In contrast, LCFF-Net-l features the highest parameter count and computational complexity, alongside superior detection accuracy. It is most appropriate for deployment on high-performance desktop computer servers.

## Results

### Dataset

Compiled by the AISKYEYE team at the Machine Learning and Data Mining Lab of Tianjin University, China, the VisDrone [32] dataset is meticulously designed to facilitate target detection, tracking, and counting tasks from aerial UAV perspectives. The dataset was curated from an extensive range of UAV-mounted camera footage captured across 14 cities in China, encompassing vast geographical expanses spanning thousands of kilometers. It comprises 261,908 frames of video footage and 10,209 still images, resulting in a total of 288 video clips. Image resolutions range from 480×360 pixels to 2000×1500 pixels, capturing diverse environments such as crowded streets, intersections, and public places. It encompasses various lighting conditions (e.g., daytime, nighttime and bright light exposure), target types (e.g., people, car and tricycle), weather conditions (e.g., sunny, cloudy and rainy), and densities (e.g., crowded or sparse scenarios).

**Fig 6:**
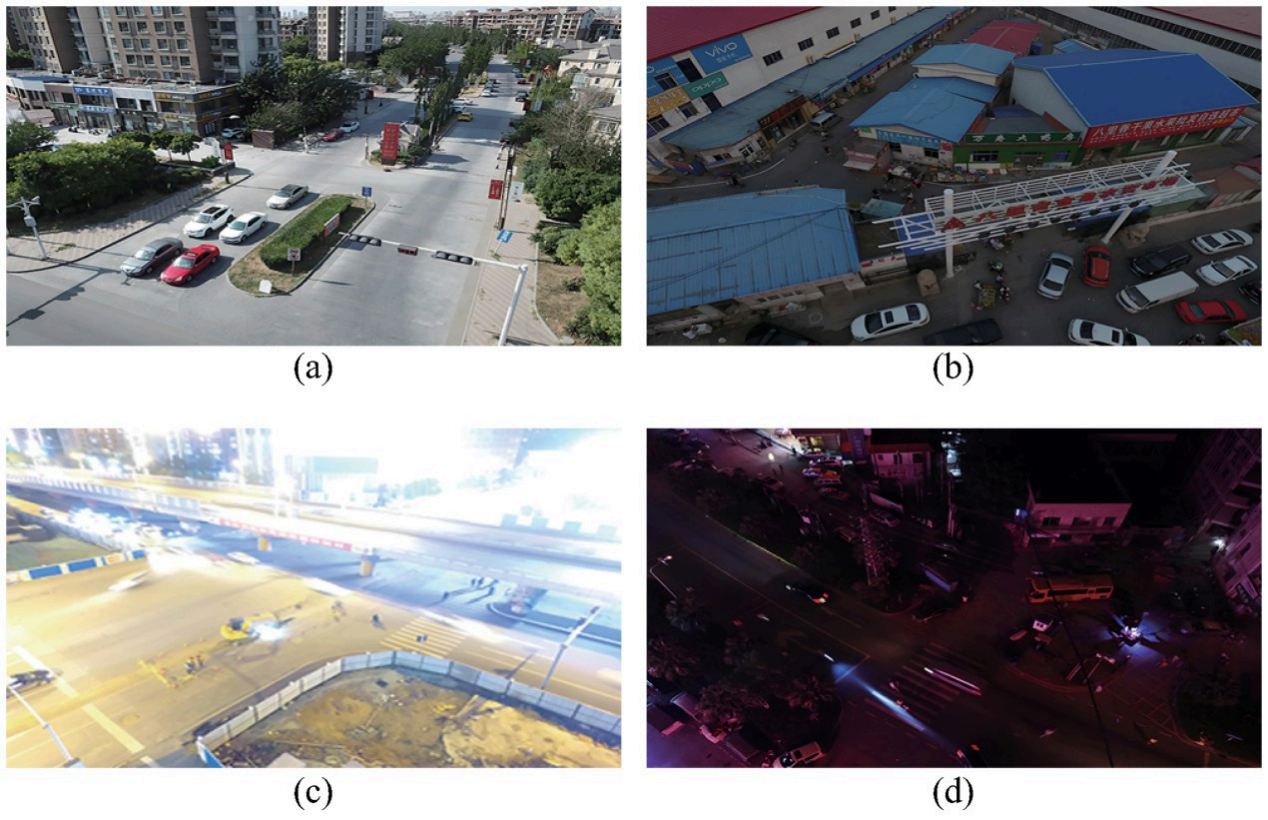
Samples of the VisDrone dataset:(a) Residential streets under normal daylight conditions;(b) dimly lit residential neighbourhoods on rainy and cloudy days;(c) Road intersections at night with strong glare;(d) Residential neighbourhoods late at night.

Over 2.6 million bounding boxes corresponding to common objects such as people, cars, motorcycles, and tricycles were manually annotated on these images, exemplifying the diversity and complexity of real-world urban environments. The perspective of these images captured from UAVs differs significantly from that of ground-level datasets (e.g., MS-COCO [20] and VOC2012 [33]) regarding camera angle, object scale, background, and weather conditions. This variability increases the dataset’s complexity, establishing it as a crucial resource for assessing the performance and robustness of computer vision models. Fig 6 presents samples from the VisDrone dataset.

To thoroughly evaluate the model’s performance, the dataset was divided into three subsets. First, the training subset, containing 6,471 images, was used to optimize the model’s parameters and facilitate robust feature learning. Second, a validation subset of 548 images was used to assess the model’s training and adjust hyperparameters, thereby preventing overfitting. Finally, the remaining 1,610 images formed the test subset, an independent set designed to evaluate the model’s generalization capability on unseen data. Table 2 presents a detailed composition of the dataset, including image and object counts for each subset and the distribution of object categories across the dataset.

**Table 2:** Labeling information of the VisDrone dataset.

Type	Car	Pedestrian	Motor	People	Van	Truck	Bicycle	Bus	Tricycle	Awning Tricycle	Image
Train	187,004	109,185	40,377	38,560	32,702	16,284	13,069	9117	6387	4377	6471
Val	14,064	8844	4886	5125	1975	750	1287	251	1045	532	548
Test	28,074	21,006	5845	6376	5771	2659	1302	2940	530	599	1610
Total	229,142	139,035	51,108	50,061	40,448	19,693	15,658	12,308	7962	5508	8629

### Experimental setup and hyperparameter configuration

The experimental setup is thoroughly outlined in Table 3, with the hyperparameters for model training specified in Table 4. Unless otherwise indicated, the experimental environment and hyperparameter configurations remain consistent across the training, testing, and validation stages. Notably, none of the experiments utilized pre-trained parameters for these networks.

**Table 3:** The experimental environment.

Parameters	Configuration
CPU	32 vCPU of AMD EPYC 9654
GPU	NVIDIA RTX 4090(24GB) × 2
Memory	120GB
Ubuntu	22.04
Python	3.12.3
Ultralytics	8.2.50
Deep learning architectures	Pytorch2.3.1+cu121, CUDA

**Table 4:** The key parameter configurations.

Parameters	Setup
Epochs	200
Image size	640 × 640
Batch size	16
workers	128
optimizer	SGD
Close mosaic	0
Patience	50
Learning rate	0.01
Momentum	0.937
Mosaic	1.0
Weight decay	0.0005

In the embedded application experiments, the Taishan Pi development board, produced by JLC Technology Group, was employed as the experimental environment. This board features an RK3566 SoC (system-on-chip). Detailed hardware and software specifications are provided in Table 5.

**Table 5:** The embedded application experimental environment.

Parameters	Configuration
CPU	Quad-Core ARM Cortex-A55
GPU	ARM G52 2EE
Memory	2GB
Ubuntu	20.04
Python	3.8.10
Ultralytics	8.2.50
Deep learning architectures	Pytorch 2.4.0

### Experiment metrics

$$\begin{array}{c} P=\frac{TP}{\left(TP+FP\right)} \end{array}$$ (7)

The experiments assess the proposed method by examining its detection accuracy, parameter footprint, and computational complexity. The evaluation metrics encompass Params (M) (millions of parameters), FLOPs (G) (giga floating-point operations), and mAP (mean average precision).

$$\begin{array}{c} R=\frac{TP}{\left(TP+FN\right)} \end{array}$$ (8)

*P* (Precision) is defined as the ratio of correctly identified targets to the total number of detected targets, as outlined in Eq (7). *R* (Recall) represents the proportion of correctly detected targets relative to the total number of actual targets, as described in Eq (8).

$$\begin{array}{c} AP=\int_{0}^{1}P(R)dR \end{array}$$ (9)

In these equations, *TP* (true positive) represents the count of accurately predicted targets, *FP* (false positive) denotes the count of incorrect predictions, while *FN* (false negative) represents the instances where actual targets were present but failed to be correctly identified.

$$\begin{array}{c} mAP=\frac{1}{k}\sum_{i=1}^{k}AP_{i} \end{array}$$ (10)

The AP (Average Precision) quantifies the area under the precision-recall curve, as formalized in Eq (9). Meanwhile, the mAP extends this concept by averaging the AP values across all categories, as indicated in Eq (10), where *K* refers to the total number of classes and *AP*_*i*_ denotes the individual average precision for each class.

The evaluation metrics utilized in this study include two distinct mean average precision measures: mAP_50_ and mAP_50–95_. For mAP_50_, a predicted bounding box is considered accurate if the IoU (Intersection over Union) between the predicted and ground truth boxes exceeds a 0.50 threshold. Conversely, mAP_50–95_ computes the average precision over a range of IoU thresholds, from 0.50 to 0.95 in 0.05 increments, with the mean precision calculated across these thresholds.

### Comparison

In this subsection, this paper compared the experimental results against algorithms from the YOLO family and several recently developed methods used the VisDrone dataset. Specifically, the algorithms included were YOLOv8 [17], YOLOv10 [19], MFFSODNet [34], LE-YOLO [24], MPE-YOLO [35], LHRYNet [36], FocusDet [37], TA-YOLO [38], APNet [39], EUAVDet [40], MFEFNet [41], BRSTD [42], SOD-YOLO-n [43], AMFEF-DETR [44], UAV-YOLO [45], MVT-B [46], and HSP-YOLO [47]. These algorithms served as the baseline for our experimental comparison.

Initially, this study presented a comparative analysis of the proposed LCFF-Net algorithm against the YOLOv8 and YOLOv10 algorithms across various model scales used the VisDrone-val dataset. The performance evaluation was based on four key metrics: *Params* (*M*), *FLOPs* (*G*), mAP_50_, and mAP_50–95_. As demonstrated in Table 6, the LCFF-Net algorithm outperforms the baseline models in terms of overall performance.

**Table 6:** Comparison of LCFF-Net’s performance with a range of other models, benchmarked on the VisDrone-val dataset.

Method	Params (M)	FLOPs (G)	mAP_50_ (%)	mAP_50–95_ (%)
YOLOv8-n	3.01	8.1	32.7	19.0
YOLOv10-n	2.27	6.5	32.8	18.7
YOLOv8-s	11.11	28.5	38.6	23.0
YOLOv10-s	7.22	21.4	39.3	23.4
YOLOv8-m	25.85	78.7	42.2	25.5
YOLOv10-m	15.32	58.9	43.0	26.0
YOLOv10-b	19.01	91.7	44.9	27.5
YOLOv8-l	43.61	164.9	43.9	27.0
YOLOv10-l	24.32	120.0	45.4	27.8
YOLOv8-x	68.13	257.4	44.9	27.8
YOLOv10-x	29.41	160.0	46.2	28.4
LCFF-Net-a	0.29	3.6	28.0	15.9
LCFF-Net-t	0.56	8.0	35.6	20.9
LCFF-Net-n	1.14	14.1	39.7	23.9
LCFF-Net-s	4.55	55.8	48.6	29.9
LCFF-Net-m	8.17	123.5	51.3	32.0
LCFF-Net-l	12.23	218.5	53.1	33.3

Specifically, when comparing the LCFF-Net-n model with the YOLOv8-s model, the LCFF-Net-n model exhibits a 2.8% enhancement in mAP_50_ and a 3.9% improvement in mAP_50–95_. Furthermore, it achieves an 89.7% reduction in model parameters and a 50.5% decrease in computational demands, significantly optimizing both efficiency and resource utilization. When assessing the LCFF-Net-m model against the YOLOv8-x model, the LCFF-Net-m shows an enhancement of 14.3% in the mAP_50_ metric and 15.1% in the mAP_50–95_ metric, accompanied by an 88.0% reduction in model parameters and a 52.0% decrease in computational requirements, further streamlining resource efficiency and computational performance.

Similarly, when comparing the LCFF-Net-n model with the YOLOv10-s model (identified as one of the more effective baseline models), the LCFF-Net-n demonstrates an improvement of 1.0% in the mAP_50_ metric and 2.0% in the mAP_50–95_ metric. Moreover, the model exhibits an 84.2% reduction in parameter count, coupled with a 34.1% decrease in computational complexity, significantly optimizing both resource efficiency and operational performance. When assessing the LCFF-Net-m model against the YOLOv10-x model, the LCFF-Net-m shows an enhancement of 11.0% in the mAP_50_ metric and 12.7% in the mAP_50–95_ metric, Accompanied by a 72.2% reduction in model parameters and a 22.8% decrease in computational demands, further enhancing both scalability and computational efficiency.

This study subsequently compared the performance of the LCFF-Net algorithm with other recently proposed algorithms using the same evaluation metrics on the VisDrone-val dataset, as detailed in Table 7. The LCFF-Net-l model, the largest in the LCFF-Net series, achieved the highest mAP_50_ and mAP_50–95_ metrics among all models. In comparison to MFEFNet, which demonstrates the highest mAP_50_ score among the baseline models, the LCFF-Net-l model achieved a 2.3% enhancement in mAP_50_ and an 11.4% improvement in mAP_50–95_, while concurrently reducing the required parameters by 63.6%. The LCFF-Net-n model, a more compact model of the LCFF-Net algorithm, outperforms MPE-YOLO with a 2.3% increase in mAP_50_ and a 3.5% improvement in mAP_50–95_. Additionally, it achieved these gains while reducing the number of required parameters by 74.1%. Furthermore, the LCFF-Net algorithm consistently outperforms the baseline model across all model sizes, exhibiting notable superiority in key metrics such as accuracy and computational efficiency.

**Table 7:** Comparison of LCFF-Net’s performance with a range of other models, benchmarked on the VisDrone-val dataset.

Method	Params (M)	FLOPs (G)	mAP_50_ (%)	mAP_50–95_ (%)
MFFSODNet [34]	4.54	-	45.5	-
LE-YOLO [24]	2.1	13.1	39.3	22.7
MPE-YOLO [35]	4.4	-	38.8	23.1
LHRYNet [36]	9.4	-	49.9	31.1
FocusDet [37]	-	-	48.7	30.4
TA-YOLO-m [38]	29.7	110.2	48.8	30.2
APNet [39]	21.3	61.9	48.7	29.8
EUAVDet-n [40]	1.34	6.9	32.9	19.2
MFEFNet [41]	33.6	-	51.9	29.9
BRSTD [42]	1.8	64.3	47.7	-
SOD-YOLO-n [43]	0.6	7.8	33.0	19.3
AMFEF-DETR [44]	35.8	142.0	41.4	24.3
UAV-YOLO [45]	7.46	17.7	38.5	-
MVT-B [46]	106.4	-	52.2	31.7
HSP-YOLO [47]	11.5	50.0	49.6	32.9
LCFF-Net-a	0.29	3.6	28.0	15.9
LCFF-Net-t	0.56	8.0	35.6	20.9
LCFF-Net-n	1.14	14.1	39.7	23.9
LCFF-Net-s	4.55	55.8	48.6	29.9
LCFF-Net-m	8.17	123.5	51.3	32.0
LCFF-Net-l	12.23	218.5	53.1	33.3

### Embedded environment deployment experiment

In the embedded environment deployment experiment, the models YOLOv8-n, YOLOv8-s, YOLOv10-n, and YOLOv10-s were selected as baseline methods for comparison with the proposed LCFF-Net-a, LCFF-Net-t, and LCFF-Net-n models. The evaluation metrics utilized for this comparison include *Params* (*M*), *FLOPs* (*G*), mAP_50_, mAP_50–95_ and the average computation time required (*Embedded* *Latency* (*s*), *Latency* *on* *server* (*s*)).

The experimental results, presented in Table 8, indicate that the LCFF-Net-a model achieves the fastest computation speed among all evaluated models. Notably, the LCFF-Net-n model is over 20% faster than YOLOv8-s and YOLOv10-s models while sustaining high accuracy. Furthermore, a comparison of computational delays between the embedded and experimental server environments reveals that the delay in the YOLOv8 and YOLOv10 series models increases by more than 5000 times in the embedded environment, whereas the LCFF-Net experiences an increase of just over 3000 times. These findings suggest that, relative to the baseline models, LCFF-Net is better suited for environments with constrained computational resources, although its delay in the embedded environment remains significantly higher than in the server setting. This underscores the substantial impact that limited computing power in embedded systems has on model calculation speed.

**Table 8:** Experimental results in the embedded environment, on VisDrone-val.

Method	Params (M)	mAP_50_ (%)	mAP_50–95_ (%)	Embedded latency (s)	Latency on server(s)
YOLOv8-n	3.01	32.7	19.0	2.24	0.00040
YOLOv8-s	11.1	38.6	23.0	5.38	0.00099
YOLOv10-n	2.27	32.8	18.7	2.11	0.00041
YOLOv10-s	7.22	39.3	23.4	5.12	0.00101
LCFF-Net-a	0.29	28.0	15.9	1.80	0.00055
LCFF-Net-t	0.56	35.6	20.9	2.95	0.00092
LCFF-Net-n	1.14	39.7	23.9	4.05	0.00122

### Ablation experiment

In the ablation experiments, YOLOv8-n was selected as the baseline model. Various structures from LCFF-Net were incrementally introduced into this baseline model, with multiple performance metrics evaluated on the VisDrone-val dataset for each modification. This approach was employed to assess the effectiveness of each structural improvement in LCFF-Net. Throughout the experiment, the experimental environment and all hyperparameter settings were maintained constant, in alignment with the experimental conditions described earlier.

As shown in Table 9, the most significant improvement in LCFF-Net is attributed to the introduction of LC-FPN. Specifically, integrating LC-FPN into the baseline model yielded a 19.0% enhancement in mAP_50_ and a 21.1% boost in mAP_50–95_, while concurrently reducing the parameter count by 34.6%. The additional structural optimizations play a pivotal role in decreasing the model’s parameter count and computational complexity, while simultaneously maintaining or improving accuracy metrics.

**Table 9:** Ablation experiment result on VisDrone-val.

LR-NET	LC-FPN	LDSCD-Head	Params (M)	FLOPs (G)	mAP_50_ (%)	mAP_50–95_ (%)
×	×	×	3.01	8.1	32.7	19.0
✓	×	×	2.51↓(16.6%)	7.7↓(4.9%)	35.3↑(8.0%)	19.4↑(2.1%)
×	✓	×	1.97↓(34.6%)	16.7↑(106.2%)	38.9↑(19.0%)	23.0↑(21.1%)
×	×	✓	2.40↓(20.3%)	7.2↓(11.1%)	32.9↑(0.6%)	19.3↑(1.6%)
✓	✓	×	1.47↓(51.2%)	16.3↑(101.2%)	40.0↑(22.3%)	23.7↑(24.7%)
✓	×	✓	1.89↓(37.2%)	6.7↓(17.3%)	32.7↑(0.0%)	19.0↑(0.0%)
×	✓	✓	1.65↓(45.2%)	14.5↑(79.0%)	39.8↑(21.7%)	23.8↑(25.3%)
✓	✓	✓	1.14↓(62.1%)	14.1↑(74.1%)	39.7↑(21.4%)	23.9↑(25.8%)

Additionally, the LCFF-Net model does not incorporate a commonly utilized attention mechanism. When addressing specific tasks, incorporating a targeted attention mechanism into the model may enhance its performance. To test this hypothesis, MLCA [48], EMA [49], CAA [50], and AA [51] were added after the SPPF module in the backbone network of the LCFF-Net model, and experiments were conducted. As shown in Table 10, although the attention mechanism was directly applied to the model’s backbone network without further optimization, the model’s performance demonstrated a partial improvement in the context of the current task. These findings indicate that incorporating a task-specific attention mechanism in the LCFF-Net model can indeed enhance its performance.

**Table 10:** Attention ablation experiment result on VisDrone-val.

Method	Params (M)	FLOPs (G)	mAP_50_ (%)	mAP_50–95_ (%)
LCFF-Net-t	0.56	8.0	35.6	20.9
+MLCA [48]	0.56	8.0	35.7 ↑(0.3%)	21.0 ↑(0.5%)
+EMA [49]	0.57	8.0	35.8 ↑(0.6%)	21.2 ↑(1.4%)
+CAA [50]	0.62	8.0	35.8 ↑(0.6%)	21.1 ↑(1.0%)
+AA [51]	0.71	8.1	36.3 ↑(2.0%)	21.3 ↑(1.9%)

### Visual analysis

To compare the object detection performance of various models visually, YOLOv8-x and YOLOv10-x are selected as baseline models for comparison against the LCFF-Net-l model proposed in this paper. The comparison is conducted across multiple criteria, including precision-recall (PR) curves, confusion matrices, heat maps of representative network layers, and direct visual assessments.

**Fig 7:**
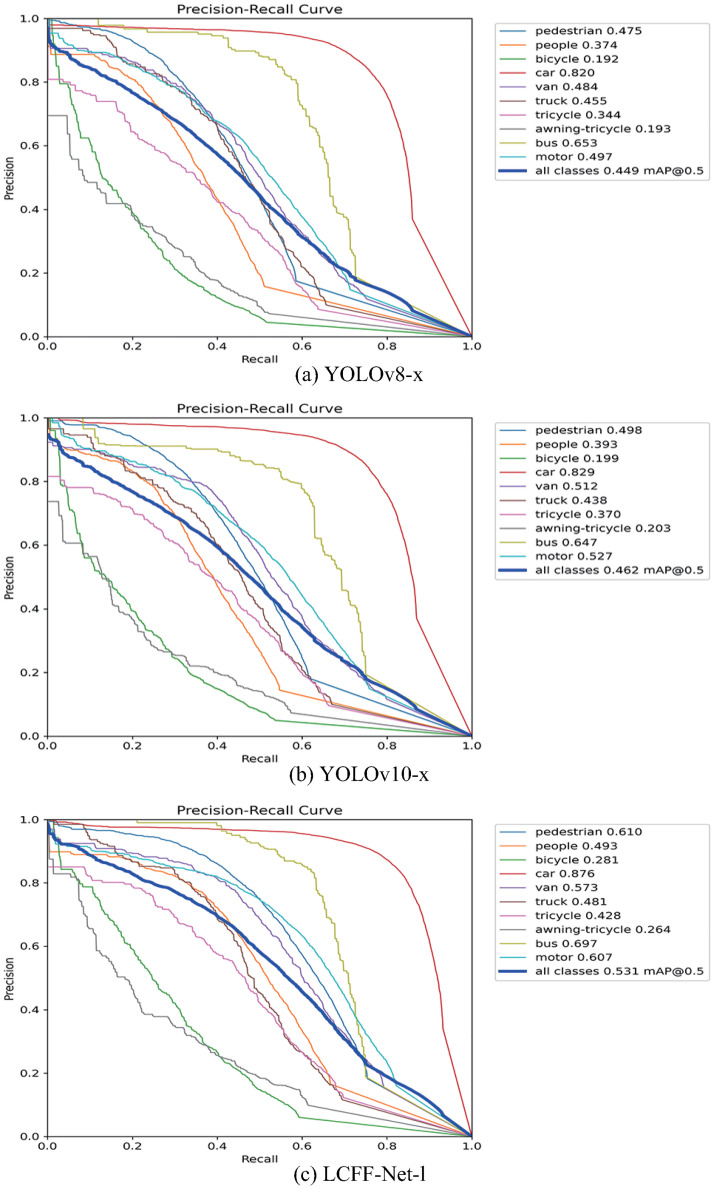
(a) Precision-Recall Curve for YOLOv8-x, (b) Precision-Recall Curve for YOLOv10-x, (c) Precision-Recall Curve for LCFF-Net-l.

Fig 7 presents the PR curves for YOLOv8-x, YOLOv10-x, and the proposed LCFF-Net-l model. The PR curve, a critical tool for evaluating model performance, is particularly useful for imbalanced datasets. It is generated by plotting precision against recall, thereby demonstrating how the model performs under varying decision thresholds.

As illustrated in Fig 7, the PR curve of the LCFF-Net-l model, compared to the YOLOv8-x and YOLOv10-x models, approaches the upper-right corner, exhibiting a larger area under the curve. This implies that the LCFF-Net-l model exhibits superior detection performance compared to the other two models. Nevertheless, despite these advancements, the detection accuracy for certain categories—specifically bicycles and awning tricycles—remains suboptimal. This observation indicates that a significant portion of these target categories is missed during the detection process.

**Fig 8:**
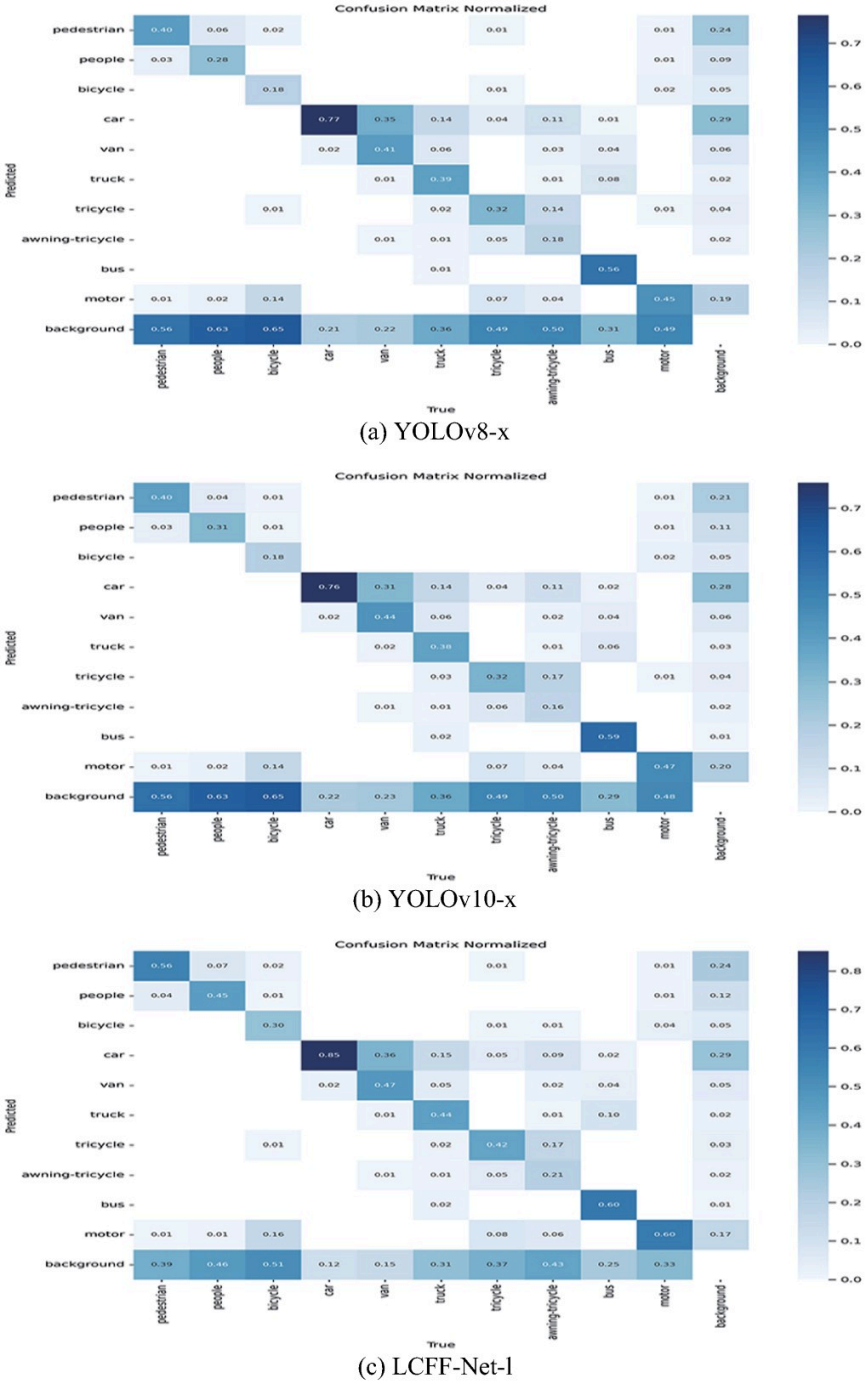
(a) YOLOv8-x Confusion Matrix, (b) YOLOv10-x Confusion Matrix, (c) LCFF-Net-l Confusion Matrix.

Fig 8 presents the confusion matrices for YOLOv8-x, YOLOv10-x, and the proposed LCFF-Net-l models to illustrate the accuracy of object detection. In these matrices, the rows denote the actual class labels, while the columns correspond to the predicted labels. The matrix values are normalized to lie within the range of 0 to 1, where the diagonal elements signify the proportion of accurate classifications for each category, and the off-diagonal elements capture the fraction of misclassifications across classes.

As illustrated in Fig 8, the confusion matrix of the proposed LCFF-Net-l model demonstrates higher intensity along the diagonal and lower intensity along the lower edge compared to the YOLOv8-x and YOLOv10-x models. This signifies that the LCFF-Net-l model exhibits superior object detection capabilities in comparison to the baseline models. Although the model achieves an overall improvement in detection accuracy, reflected in a lower missed detection rate across most categories, it continues to exhibit certain errors in specific categories where the baseline models do not. This underscores the presence of residual challenges and suggests potential for further refinement in detection accuracy.

**Fig 9:**
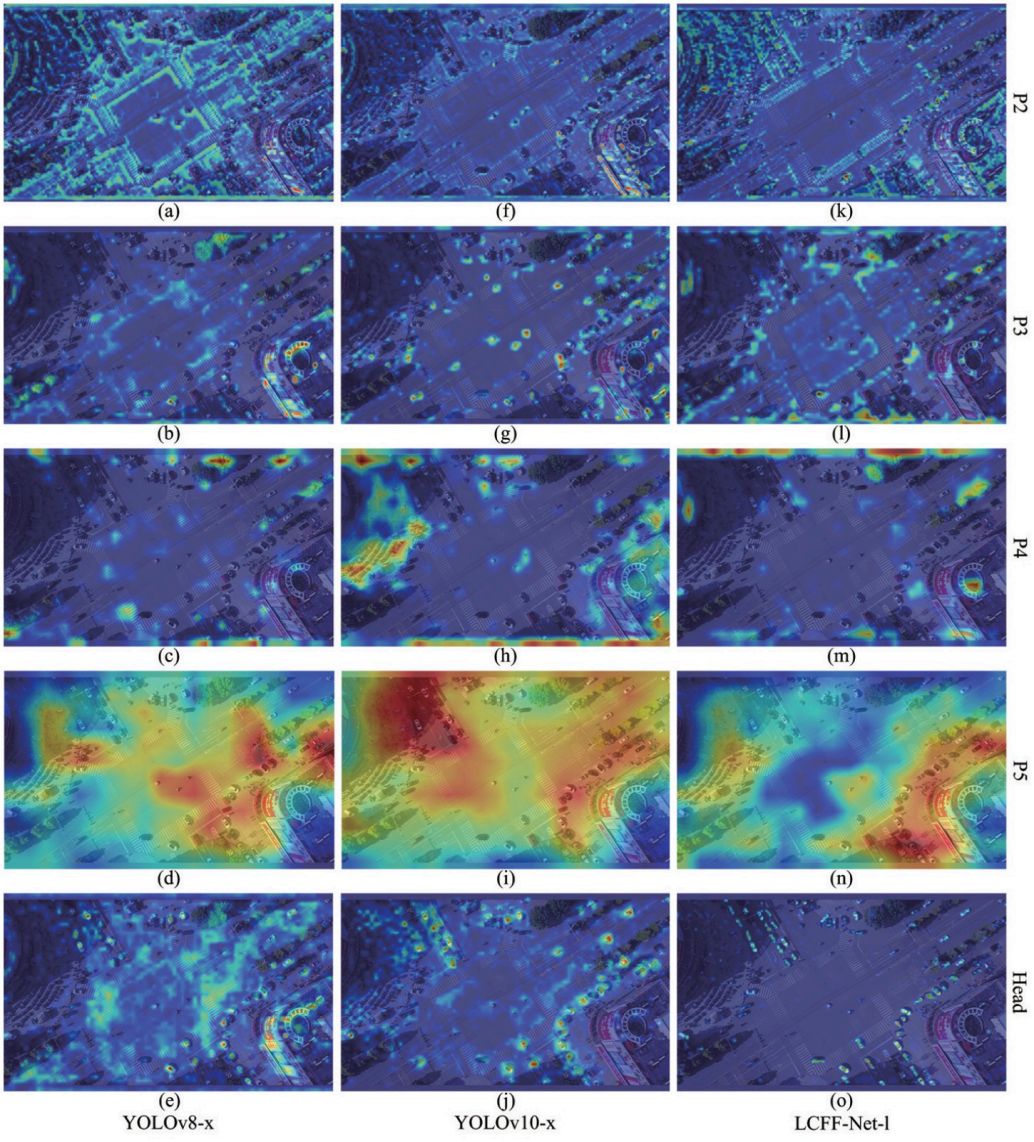
Comparative visualization of Grad-CAM heat maps for the prediction process between YOLOv8-x, YOLOv10-x, and the proposed LCFF-Net-l models representative network layers (i.e., P2, P3, P4, P5, and Head). (a–e) show the Grad-CAM heat maps for the prediction process of the YOLOv8-x model; (f–j) show the Grad-CAM heat maps for the YOLOv10-x model; and (k–o) present the Grad-CAM heat maps for the LCFF-Net-l model.

Fig 9 presents the Grad-CAM heatmap visualizations of representative network layers (P2, P3, P4, P5, and the Head layer) in the YOLOv8-x, YOLOv10-x, and the proposed LCFF-Net-l models. As illustrated in Fig 9(a)–9(j), both the YOLOv8-x and YOLOv10-x models tend to focus on features with a broader scope, thereby failing to adequately capture the features of tiny objects. In contrast, Fig 9(k)–9(o) demonstrate that the LCFF-Net-l model significantly improves the focus on tiny objects, highlighting their key features with superior accuracy and comprehensiveness. These visual heatmaps clearly indicate that the LCFF-Net-l model surpasses the YOLOv8-x and YOLOv10-x baseline models in accuracy when detecting tiny targets. The Grad-CAM heatmap visualizations provide critical insights into the model’s decision-making process, further corroborating the exceptional performance of the LCFF-Net-l model in accurately detecting small-scale targets.

**Fig 10:**
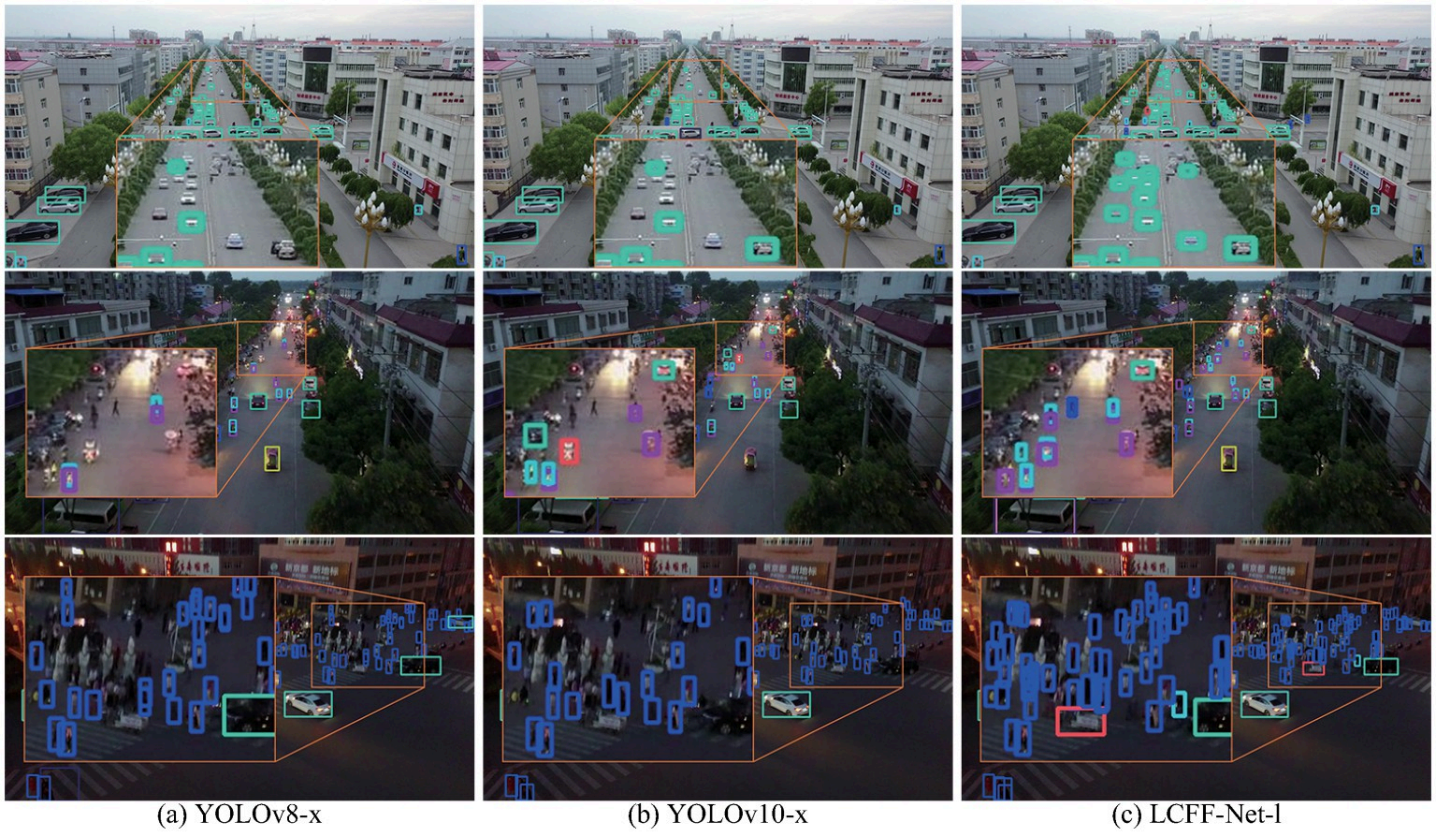
Comparative visualization of detection outcomes on samples from the VisDrone-val dataset: (a) Predictions by YOLOv8-x, (b) Predictions by YOLOv10-x, and (c) Predictions by LCFF-Net-l.

**Fig 11:**
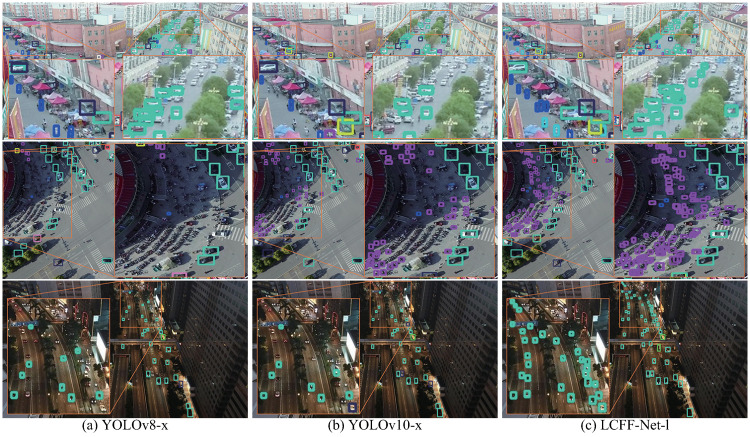
Comparative visualization of detection outcomes on samples from the VisDrone-test dataset: (a) Predictions by YOLOv8-x, (b) Predictions by YOLOv10-x, and (c) Predictions by LCFF-Net-l.

To assess the robustness and effectiveness of the LCFF-Net-l model in challenging scenarios, its capacity to detect tiny targets across diverse and complex environments was rigorously analyzed and compared against the performance of the YOLOv8-x and YOLOv10-x models. The significant discrepancies in detection results between models are highlighted in orange boxes in the accompanying Figs, with enlarged views provided for clarity. Six complex scenes were selected from the VisDrone-val (Fig 10) and VisDrone-test (Fig 11) datasets, incorporating diverse lighting conditions (e.g., daytime, nighttime, glare, and shadows), target types (pedestrians, vehicles, bicycles, and tricycles), and environmental settings (sparse versus crowded areas), which are common in UAV aerial imagery yet notoriously difficult to detect accurately.

As demonstrated in Figs 10a, 10b, 11a and 11b, the YOLOv8-x and YOLOv10-x models tend to overlook tiny objects in distant, crowded, and dimly lit environments, leading to challenges in accurately detecting these objects. Conversely, the LCFF-Net-l model proposed in this study facilitates more efficient and accurate detection of tiny objects across a range of challenging conditions, such as distant, densely populated, high-glare, or low-light environments.

In summary, the findings highlight the LCFF-Net model’s versatility and efficacy in detecting tiny aerial targets within intricate urban landscapes. The model consistently achieves high accuracy and computational efficiency, even under demanding conditions, underscoring its robustness and adaptability across a wide spectrum of real-world scenarios.

## Discussion and conclusion

This paper presents LCFF-Net, a lightweight cross-scale feature fusion network designed specifically for the detection of tiny targets in UAV aerial imagery. The algorithm addresses key challenges in UAV image target detection, such as varying lighting conditions, complex environments, and tiny target sizes. To mitigate these challenges, a Lightweight Feature Extraction Convolutional Block (LFECB) was designed and a Lightweight Feature Extraction Reparameterized Efficient Layer Aggregation Network (LFERELAN) was developed. This structure efficiently extracted target features while reducing computational costs. Furthermore, the LC-FPN and LR-NET architectures, which enhance the backbone and neck structures of baseline model, were introduced respectively. These modifications enable low-cost fusion of multi-level network features and bottom-layer features rich in spatial information, significantly improving the model’s ability to detect tiny objects. Additionally, the Lightweight Detail-Enhanced Shared Convolution Detection Head (LDSCD-Head) was designed to improve the detection head of baseline model. This detection head enables efficient multi-scale feature fusion and sharing, optimizing the utilization of information across various scales while substantially minimizing the model’s parameter complexity and computational overhead. Empirical results on the VisDrone-val dataset demonstrated that the proposed LCFF-Net-l model outperforms the baseline across both the mAP_50_ and mAP_50–95_ performance metrics. The smaller LCFF-Net-n model improves mAP_50_ by 2.8% and mAP_50–95_ by 3.9% compared to the baseline, while reducing model parameters by 89.7% and computational costs by 50.5%. Furthermore, the LCFF-Net algorithm demonstrates faster computational speeds in embedded environments compared to the baseline. Notably, our network improvement method does not incorporate the widely-used attention mechanism, suggesting potential for further task-specific optimizations.

The LCFF-Net algorithm includes models of varying scales. The smaller models are optimized for embedded deployment under constrained computational resources, whereas the larger models are designed for desktop computing platforms, making them suitable for different application scenarios. While LCFF-Net has been optimized for embedded environments, particularly in extreme conditions, its performance still shows potential for further enhancement. Future work could focus on refining the model’s computational efficiency to improve its robustness in challenging scenarios. Additionally, incorporating task-specific attention mechanisms may offer further performance gains with minimal computational overhead.

## Supporting information

S1 File The VisDrone dataset referenced in this study is publicly accessible and can be retrieved from https://github.com/VisDrone/VisDrone-Dataset. (DOCX)

S2 File The source code for the LCFF-Net model is available at https://github.com/Tdzdele/LCFF-Net. (DOCX)
