# Supplementary material for: LCFF-Net: A lightweight cross-scale feature fusion network for tiny target detection in UAV aerial imagery
Source: PLoS One. 2024 Dec 19;19(12):e0315267. doi: 10.1371/journal.pone.0315267 (PMC11658636; doi:10.1371/journal.pone.0315267)
Supplement: S1 File — (DOCX) [file pone.0315267.s001.docx]

S1 File. The VisDrone dataset referenced in this study is publicly 539

accessible and can be retrieved from 540

https://github.com/VisDrone/VisDrone-Dataset.
