# Supplementary material for: LCFF-Net: A lightweight cross-scale feature fusion network for tiny target detection in UAV aerial imagery
Source: PLoS One. 2024 Dec 19;19(12):e0315267. doi: 10.1371/journal.pone.0315267 (PMC11658636; doi:10.1371/journal.pone.0315267)
Supplement: S2 File — (DOCX) [file pone.0315267.s002.docx]

S2 File. The source code for the LCFF-Net model is available at 542

https://github.com/Tdzdele/LCFF-Net.
